# Frequent blood feeding enables insecticide-treated nets to reduce transmission by mosquitoes that bite predominately outdoors

**DOI:** 10.1186/s12936-016-1195-8

**Published:** 2016-03-10

**Authors:** Tanya L. Russell, Nigel W. Beebe, Hugo Bugoro, Allan Apairamo, Weng K. Chow, Robert D. Cooper, Frank H. Collins, Neil F. Lobo, Thomas R. Burkot

**Affiliations:** Australian Institute of Tropical Health and Medicine, James Cook University, Cairns, QLD 4870 Australia; School of Biological Sciences, University of Queensland, St. Lucia, Brisbane, QLD 4068 Australia; CSIRO, Dutton Park, Brisbane, QLD 4102 Australia; National Vector Borne Disease Control Programme, Ministry of Health, Honiara, Solomon Islands; Australian Army Malaria Institute, Gallipoli Barracks, Enoggera, Brisbane, 4052 Australia; Department of Biological Sciences, Eck Institute for Global Health, University of Notre Dame, Notre Dame, IN 46556 USA

**Keywords:** *Anopheles farauti*, Solomon Islands, Bionomics, Mark-release-recapture, Feeding cycle, Seasonality, Biting profile

## Abstract

**Background:**

The effectiveness of vector control on malaria transmission by long-lasting insecticidal nets (LLINs) and indoor residual spraying (IRS) depends on the vectors entering houses to blood feed and rest when people are inside houses. In the Solomon Islands, significant reductions in malaria have been achieved in the past 20 years with insecticide-treated bed nets, IRS, improved diagnosis and treatment with artemisinin combination therapies; despite the preference of the primary vector, *Anopheles farauti*, to feed outdoors and early in the evening and thereby avoid potential exposure to insecticides. Rational development of tools to complement LLINs and IRS by attacking vectors outdoor requires detailed knowledge of the biology and behaviours of the target species.

**Methods:**

Malaria transmission in Central Province, Solomon Islands was estimated by measuring the components comprising the entomological inoculation rate (EIR) as well as the vectorial capacity of *An. farauti*. In addition, the daily and seasonal biting behaviour of *An. farauti*, was examined and the duration of the feeding cycle was estimated with a mark-release-recapture experiment.

**Results:**

*Anopheles farauti* was highly exophagic with 72 % captured by human landing catches (HLC) outside of houses. Three-quarters (76 %) of blood feeding on humans was estimated to occur before 21.00 h. When the hourly location of humans was considered, the proportion of exposure to mosquito bites on humans occurring indoors (π_i_) was only 0.130 ± 0.129. Peak densities of host seeking *An. farauti* occurred between October and January. The annual EIR was estimated to be 2.5 for 2012 and 33.2 for 2013. The length of the feeding cycle was 2.1 days.

**Conclusions:**

The short duration of the feeding cycle by this species offers an explanation for the substantial control of malaria that has been achieved in the Solomon Islands by LLINs and IRS. *Anopheles farauti* is primarily exophagic and early biting, with 13 % of mosquitoes entering houses to feed late at night during each feeding cycle. The two-day feeding cycle of *An. farauti* requires females to take 5–6 blood meals before the extrinsic incubation period (EIP) is completed; and this could translate into substantial population-level mortality by LLINs or IRS before females would be infectious to humans with *Plasmodium falciparum* and *Plasmodium vivax*. Although *An. farauti* is primarily exophagic, the indoor vector control tools recommended by the World Health Organization (LLINs and IRS) can still provide an important level of control. Nonetheless, elimination will likely require vector control tools that target other bionomic vulnerabilities to suppress transmission outdoors and that complement the control provided by LLINs and IRS.

## Background

The basic reproductive number (R_0_) [1] is determined by the intensity of malaria transmission which depends largely on the parameters comprising vectorial capacity [2, 3] (the human biting density, proportion of blood-meals on humans and the mosquito life expectancy). The vector life expectancy, in turn, is a function of its survivorship per feeding cycle and the length of the feeding or gonotrophic cycle. The effectiveness of vector control depends on where and when a vector seeks human blood meals (which is determined, in part, by the location of humans). These parameters vary by species, both geographically and temporally, and will determine the effectiveness of vector control strategies implemented across different seasons and locations.

The Solomon Islands is currently undertaking country-wide intensified malaria control with the goal of malaria elimination in targeted provinces. Malaria transmission is predominantly by *Anopheles farauti.* The vector control strategies are those recommended by the World Health Organization’s Malaria Policy Advisory Committee (WHO MPAC)—universal distribution of long-lasting insecticidal nets (LLINs) and indoor residual spraying (IRS) in limited areas [4]. Exposure of vectors to the insecticides occurs when mosquitoes enter houses late at night while seeking a blood meal (LLINs) or when resting after blood feeding (IRS) [5, 6]. Although *An. farauti* varies in its degree of anthropophagy across Melanesia, it is highly anthropophagic in the Solomon Islands [7]. In the Solomon Islands, *An. farauti* displays behavioural resistance to insecticides by feeding mostly outdoors and early in the evening [8]. *Anopheles farauti* first shifted its behaviour to feed early in the evening when people were outdoors in response to the DDT spray campaigns in the 1970s, thus avoiding the insecticide [9, 10]. This behavioural shift was one reason that the original Malaria Eradication Programme (MEP) of the early 1970s failed [11] and, malaria cases surged throughout the Solomon Islands until insecticide treated nets (ITNs) and LLINs were introduced in 1992–1993, and 2005, respectively [12]. This insecticide avoidance behaviour appears to be maintained by the widespread use of LLINs, as recent surveys show that this early outdoor biting behaviour still persists in at least three other *An. farauti* populations in the Solomon Islands [13–15].

Despite the challenge of behavioural resistance in *An. farauti*, there have been significant reductions in malaria achieved in the Solomon Islands in the past 20 years with ITNs, IRS and improved anti-malarials. However, malaria elimination remains, perhaps, an insurmountable challenge with these available intervention tools. New vector control interventions are needed to complement the indoor killing of LLINs and IRS by attacking outdoor feeding or other behavioural vulnerabilities of *An. farauti* [16, 17]. Rational development of such tools requires detailed knowledge about the biology and behaviours of vectors. The isolated island populations of *An. farauti* display variability in their night biting profile, blood feeding patterns and the degree of endophily, likely the result of restricted gene flow among island populations [18]. In this paper, a number of key vector parameters were measured for *An. farauti*, in Central Province, Solomon Islands to determine potential behavioural vulnerabilities for vector control. These parameters were the daily and seasonal biting behaviour, the time and location (indoors or outdoors) of blood feeding and the length of the feeding cycle.

## Methods

### Study site

**Fig. 1:**
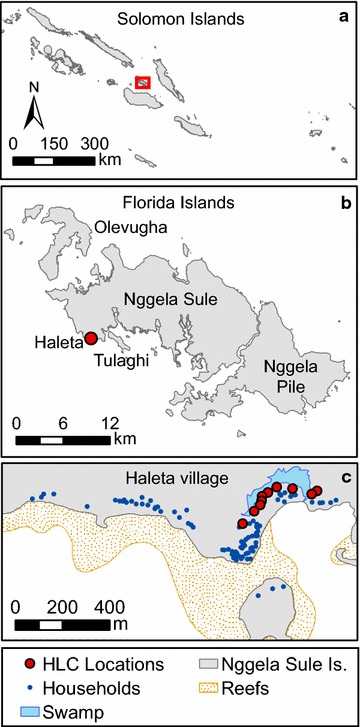
Map of the Solomon Islands (**a**) showing Haleta village on Nggela Sule Island in Central Province (**b** −9°5′56″S, 160°6′56″E) as well as the layout of the village (**c**)

The study was conducted in Haleta village on Ngella Sule Island in Central Province (−9°5′56″S, 160°6′56″E; Fig. 1) where malaria transmission is hypoendemic [19]. This rural coastal village is bounded by the ocean to the south, with high ground of ≈360 m elevation on the north. This community of 470 people live in 107 houses constructed predominantly of bamboo walls or woven palm fronds with thatched roofs and open eaves (Bed net census, 2010, Solomon Islands Ministry of Health, Unpublished data). Domestic animals consist of pigs (predominantly housed in pens), chickens, dogs and cats. The climate of the region is continuous hot/wet with a median annual rainfall of 2837 mm (based on 43 years of data collected at the provincial capital Tulagi approximately 10 km from Haleta village) [20]. While rain falls throughout the year, there is higher precipitation from January to March (mean monthly rainfall of 344 mm), with relatively less rain between April and December (mean monthly rainfall of 200 mm). The mean daily coastal temperature ranges between 24 and 30 °C with an annual mean of 26 °C.

### Mosquito sampling and processing

Anopheline mosquitoes were sampled by human landing catches (HLC) [21]. Village collectors captured mosquitoes that landed on their exposed legs and feet with a mouth aspirator at designated collection stations in Haleta village. Mosquitoes were held in individual waxed paper cups by hour and location of collection (geographic location within the village or indoors/outdoors). Numbers of *An. farauti* caught per hour and location per collector were recorded based on morphological examination [22] prior to dissecting subsamples for parity determination and spermatheca insemination [23]. Parity was assessed by dissecting the ovaries, drying on a glass microscope slide and examining under 100–200 times magnification for the presence or absence of skeins at the end of the tracheoles [23]. The insemination status of female *An. farauti* was assessed by dissecting and rupturing the spermatheca under a cover slip and examining under 400 times magnification for spermatozoa [23]. All specimens were preserved in 100 % ethanol and a subsample subjected to subsequent species identification analysis using the Internal Transcribed Spacer Region II of the ribosomal DNA (ITS2) [24] and detection of *Plasmodium* DNA in heads and thoraces by nested PCR [25].

### All night biting profile

The indoor and outdoor biting profile of *An. farauti* was estimated from five households from 18.00 to 06.00 h from the 24th to the 28th of July 2012 by HLC. Mosquitoes were collected by hour and the households were separated by a distance of ≥20 m with collectors working in pairs, one indoors the other outdoors 10 m away. The collectors were systematically rotated between working the early and late shifts on each night.

The biting behaviour of *An. farauti* was analysed to estimate endophagy, nocturnal biting and human contact indoors (π_i_). Endophagy or the propensity to bite indoors was defined as the total number of *An. farauti* collected indoors divided by the total of indoor plus outdoor *An. farauti* collected. The ability to obtain a blood meal on humans indoors (nocturnal activity) was calculated as the total number of bites indoors plus outdoors during sleeping hours (21.00–05.00 h) divided by the total during the entire night. The analysis was extended to calculate the proportion of human contact with mosquito bites occurring indoors (π_i_) (see [26] for detailed formulas). To determine this, the number of people outdoors in the HLC area was counted hourly from 18.00 to 06.00 h each night for 14 consecutive nights beginning on 23rd Nov 2011. The number of people indoors for each hour was calculated as the difference between the hourly outdoor count and the mean number of occupants seen outdoors at 18.00 h.

### Seasonality of *Anopheles farauti*

Biting densities of *An. farauti* were estimated by HLC between August 2011 and February 2014 from 18.00 to 00.00 h for a minimum of five nights each month by 10 village collectors working outdoors distributed along the village (Fig. 1). As the majority of biting occurred from 18.00 to 00.00 h, this time period was selected as a time and cost effective measure for estimating anopheline density and seasonality. Samples of *An. farauti* were dissected to determine parity (six occasions: November 2011, February, May, July, August, and November 2012) and insemination status (three occasions: November 2011, February and May 2012). PCR assays were used to identify a subset of specimens species [24] and identify infections of *Plasmodium* sporozoites [25].

For 2012 and 2013, the annual entomological inoculation rate (EIR) was calculated using the equation: EIR = S × B × 365 [27, 28]. Where, S is the sporozoite rate (defined as the number of mosquitoes with malaria specific DNA detected in the head and thorax/no. of mosquitoes tested), and B is the annual human biting rate (mean number of mosquitoes collected per collector per night/calibration factor). As mosquitoes were only collected from 18.00 to 00.00 h the estimated biting rate was adjusted to account for the proportion of *An. farauti* that fed after midnight; using a calibration factor of 0.93 to estimate the all night biting rate (see Results).

### Duration of the gonotrophic cycle

Freshly blood-fed *An. farauti* from HLC were placed individually into 70 ml specimen jars with damp cotton-wool covered with filter paper as an oviposition substrate. The top of each jar was covered with netting and damp cotton-wool to maintain high humidity. The containers were held at ambient temperature and exposed to normal day/night light regimes. Hourly examination of the containers for eggs commenced at dusk (18.00 h) 43 h after blood-feeding. The hour in which eggs were laid was recorded.

### Duration of the feeding cycle by mark-release-recapture experiment

The length of the feeding cycle (defined as the period between two consecutive blood-meals) for *An. farauti* was estimated from a mark-release-recapture experiment using mosquitoes captured by HLC from 29th November to 9th December 2012. Mosquitoes were collected from 18.00 to 00.00 at 16 outdoor HLC stations positioned throughout the village (Fig. 1). Blood fed mosquitoes were placed into plastic 250 ml cups covered with netting, each cup containing a maximum of 100 mosquitoes. A small amount of fluorescent powder (BioQuip Products, Inc. California, USA and Glow Paint Industries, Queensland, Australia) was sifted through the netting into the cup; a fine tipped transfer pipette was use to aerosolise the powder which coated the mosquitoes. The effectiveness of this procedure was checked by examining the mosquitoes in each cup with a LED UV torch (400 nm wavelength) to ensure that they were adequately marked with the powder. The mosquitoes were released between 00.00 h and 01.00 h on the night of collection from a single outdoor location. The distance from the release site to the most distant HLC collection station was 190 m. Mosquitoes were marked on nights 1, 2 and 3 using a different colour (blue, pink, and yellow) fluorescent powder each night. On nights 2 through 11, all captured *An. farauti* were visually checked for fluorescent dust using a UV torch. Recaptured marked mosquitoes and unmarked mosquitoes which were not released were stored for species identification.

The mean length of the feeding cycle (U) was estimated as:

$$U = \frac{{(2 \times R_{2} + 3 \times R_{3} )}}{{(R_{2} + R_{3} )}}$$

where R represents the number of mosquitoes recaptured on day *i* after release [29, 30].

### Statistical analysis

The data was compiled in a series of tables which detailed the results of: (1) mosquito collections, (2) dissections, (3) molecular analyses, (4) mark-release-recapture releases, and (5) oviposition [31]. Statistical differences in endophagy (indoor versus outdoor biting) and nocturnal biting (sleeping hours were 21.00–05.00 h) were compared with generalized linear models (GLMs) with a negative binomial distribution. The temporal change in the biting rate and the proportion parous were analysed with GLMs with a negative binomial and binomial distributions, respectively. All analyses were conducted using the *R* package V3.1.2 [32].

### Ethics

Ethical approval for the study was obtained from the National Health Research and Ethics Committee, Solomon Islands (02-05-2011), the James Cook University Human Research Ethics Committee, Australia (H4122) and the University Hospitals Case Medical Centre Institutional Review Board for Human Investigation, USA (05-11-11). Collectors were selected and trained from residents of Haleta after obtaining informed consent. Only village adults who likely have some immunity to malaria were asked to participate in the landing catches and were instructed to capture the mosquitoes before they bite and all took malaria prophylaxis. To estimate the duration of the feeding cycle by mark-release-recapture, mosquitoes were offered a human blood meal from one of the listed authors who was taking malaria prophylaxis prior to release.

## Results

In Haleta village, 21,619 female anophelines were collected by HLC. All specimens were morphologically *An. farauti s.l.* A subset of the specimens (n = 1315) were confirmed as *An. farauti s.s.* by molecular analysis (with samples selected across the longitudinal dataset).

### All night biting profile of *Anopheles farauti*

**Fig. 2:**
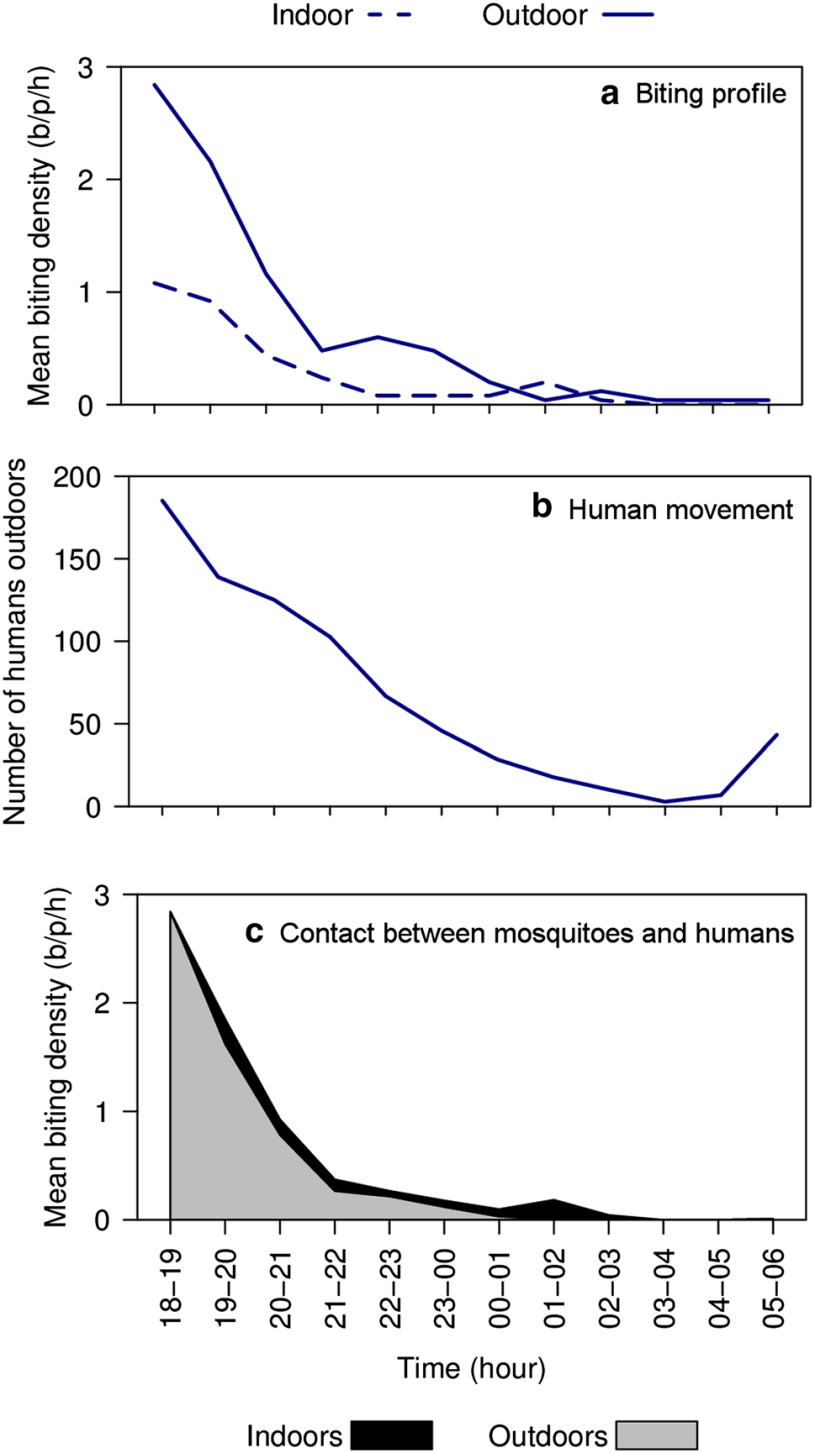
The hourly indoor and outdoor profile of *Anopheles farauti* biting (**a**) and the number of humans outside of houses throughout the night (**b**) in Haleta village, Central Province, Solomon Islands. The stacked *line graph* (**c**) is the estimated contact between humans and mosquitoes, which considers the movement pattern of people by weighting the indoor and outdoor biting rates throughout the night by the proportion of humans that are typically indoors or outdoors at each time period [41, 42]. *Note* b/p/h = bites/person/hour

*Anopheles farauti* was highly exophagic (*β* = 0.953, se = 0.197, *p* < 0.0001), with the proportion of endophagy estimated as 0.28 ± 0.03 (mean proportion indoors ± se; Fig. 2a). The nocturnal biting activity (proportion biting during sleeping hours [21.00–05.00 h]) of *An. farauti* was 0.239 ± 0.025 (proportion nocturnal ± se). Significantly more biting occurred outside of sleeping hours (*β* = 1.625, se = 0.187, *p* < 0.0001) with 76 % of the overall biting occurring before 21.00 h and 93 % before midnight (Fig. 2b). After adjusting for human behaviour (location of people indoors or outdoors over the night), the estimated biting rate for an unprotected person (B_u_) was 6.8 bites per night and the proportion exposed to mosquito bites indoors (π_i_) was only 0.130 ± 0.129 (Fig. 2c).

### Seasonality of *Anopheles farauti*

**Fig. 3:**
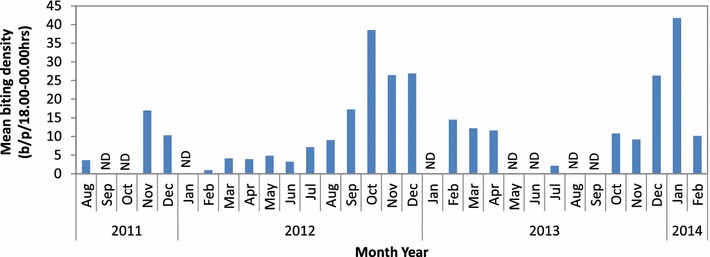
Monthly biting rate for *Anopheles farauti* in Haleta village, Central Province, Solomon Islands, estimated using human landing catch from 18.00 to 00.00 h. *Note* *ND* no data

The density of host seeking *An. farauti* varied temporally (*β* = 0.078, se = 0.002, *p* < 0.0001) with the highest densities occurring between October and January, reaching ≈40 bites per person from 18.00 to 00.00 h in October 2012 and January 2014 (Fig. 3). The average human biting rate of *An. farauti* was 14.81 bites/person/night (b/p/n). The sporozoite rate in *An. farauti* was 0.0047 based on the analyses of 4707 *An. farauti* heads and thoraxes for *Plasmodium* DNA. The overall EIR was estimated to be 25.3 infective bites/person/year (ib/p/y; Table 1). The EIR varied annually from 2.5 ib/p/y in 2012 to 35.7 ib/p/y in 2013. The overall parity rate of *An. farauti* was estimated to be 0.59 parous (439/739, for the period November 2011 to December 2012). Parity was temporally influenced (*β* = −0.338, se = 0.044, *p* < 0.0001) with monthly parity estimates ranging between 0.41 and 0.76; with parity rates higher in February (0.73; n = 184), May (0.57; n = 118) and July (0.62; n = 43) and lowest in the latter half of the year in August (0.44; n = 133) and November (0.41; n = 131). The majority, 96 %, of host-seeking *An. farauti* were inseminated (n = 344/358).

**Table 1:** The estimated malaria transmission intensity attributable to *Anopheles farauti* in Haleta village, Central Province, The Solomon Islands

Time	Sporozoite rate (n positive)				All night biting rate (b/p/18.00–06.00 h)^a^	Daily EIR (ib/p/d)^b^	Annual EIR (ib/p/y)^c^
	Total tested	*P. falciparum*	*P. vivax*	Overall			
Nov 2011	207	0.0000 (0)	0.0000 (0)	0.0000 (0)	15.16	0.000	
2012	2062	0.0000 (0)	0.0005 (1)	0.0005 (1)	13.98	0.007	2.5
2013	1907	0.0026 (5)	0.0052 (10)	0.0073 (14)^d^	13.33	0.098	35.7
Jan–Feb 2014	531	0.0038 (2)	0.0113 (6)	0.0132 (7)^d^	27.90	0.368	
Overall	4707	0.0015 (6)	0.0036 (17)	0.0047 (22)^d^	14.81	0.069	25.3

^a^All night biting rate was calculated with a calibration factor of 93 % biting before midnight. This figure was calculated from the biting profile presented in the first section of this paper

^b^Daily EIR [infective bites per person per day (ib/p/d)] = sporozoite rate × biting rate (18.00–06.00 h)

^c^Annual EIR [infective bites per person per year (ib/p/y)] = sporozoite rate × biting rate (18.00–06.00 h) × 365

^d^These sample periods include mixed *P. falciparum* and *P. vivax* infections (one from 2013, one from Jan–Feb 2014 and thus two mixed infections overall)

### Duration of the gonotrophic cycle

The time from blood engorgement to oviposition was recorded for 145 *An. farauti*. Of these, 44.1 % (n = 64) of *An. farauti* laid eggs on the second night after blood-feeding, 46.2 % (n = 67) laid eggs on the third night and the remainder (9.7 %; n = 14) laid on the forth night. The average interval from blood feeding to oviposition was 61.2 ± 1.1 h (n = 111) or 2.6 days. The length of the gonotrophic cycle ranged from 43 to 83 h.

### Duration of the feeding cycle

**Fig. 4:**
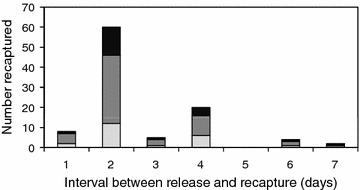
The feeding cycle length of *Anopheles farauti* examined by a mark-release-recapture experiment, expressed as a frequency histogram of the interval of time between release and recapture for each individual mosquito. Note: the different *colours* represent the information from each of the three events when mosquitoes were released

During this experiment, 3891 anophelines were captured by HLC and identified morphologically as *An. farauti s.l.*, with 100 % of a subset being molecularly identified as *An. farauti s.s.* (n = 189). To estimate the length of the feeding cycle, 1751 blood-fed female *An. farauti* of unknown chronological age were marked with fluorescent dust (a different colour on each night) and released (282 on night 1, 266 on night 2 and 203 on night 3). Subsequently, 105 marked *An. farauti* were recaptured (a recapture rate of 14 %). The interval between release and recapture (the length of the feeding cycle) was 2.1 days (Fig. 4). Three feeding cycles of two days duration are clearly evident after the mosquitoes were released. The majority, 82 %, of mosquitoes sought blood meals two nights after their previous blood meal, with 11 and 7 % seeking blood meals at 1 and 3 night intervals, respectively.

## Discussion

The effectiveness of vector control is a function of both mosquito and human behaviours. For LLINs and IRS, the degree to which the vector feeds or rests indoors (i.e., how endophagic or endophilic) as well as the frequency at which the vector blood feeds will largely determine the proportion that survive for the duration of the extrinsic incubation period. Indoor feeding and resting are determined, in large part by the location of humans (indoors or outdoors) when mosquitoes are seeking blood meals (e.g., mosquitoes seeking human blood meals earlier in the evening are more likely to feed on humans outdoors when few people are inside houses). The duration of peak mosquito density is important for the selection and timing of the application of insecticides used in IRS (as different insecticides and formulations vary in their effective half-life).

Most populations of *An. farauti* in the Solomon Islands bite outdoors and early in the evening. Previously reported π_i_ values (the proportion of feeds on humans taking place indoors) were 0.314 for Guadalcanal Province in 2007-08 [15]^1^ to 0.368–0.570 for Temotu Province in 2008–2010 [13] with the highest value recorded in Isabel Province in 2009 (0.546) [14]. The lowest proportion of bites on humans indoors for *An. farauti* was found in this study in Haleta village on Ngella Sule, Central Province, with only 13 % of human feeds indoors. This island was designated as a “problem area” during the original malaria eradication programme [10], which is understandable as the early outdoor feeding of *An. farauti* found in this study would minimize exposure to the insecticides used in IRS and ITNs and limit the effectiveness of the interventions.

The terms gonotrophic and feeding cycle are often used interchangeably despite the fact that they are, in fact, describing slightly different time intervals (i.e., the period between successive oviposition and blood feeding events, respectively). Mark-release-recapture experiments using HLC estimated the feeding cycle length whereas the gonotrophic cycle length was estimated by measuring the duration between blood feeding and oviposition of mosquitoes held under field laboratory conditions. Feeding cycle length estimates from mark-release-recapture, for all anopheline species range from 2 to 4 days [33, 34]. The feeding cycle length for *An. farauti* in Central Province is one of the shortest recorded at 2.1 days, but is comparable with previous estimates for this species from Guadalcanal Province, Solomon Islands [35] and Madang Province, Papua New Guinea [29, 30, 36] which ranged between 2 and 3 days. The feeding cycles among malaria vectors in different villages in Madang, Papua New Guinea were 2.7–3.7 days for *Anophelespunctulatus*, 2.4–3.2 days for *Anopheles koliensis* and 2.1–3.0 days for *An. farauti* [30]. The local environment was found to exert a greater influence on the duration of the feeding cycle than the species of mosquito, with permanent pool breeders having a shorter cycle then temporary pool breeders. If extensible to the Solomon Islands, the environmental conditions in the coastal villages where *An. farauti* is found would have been predicted to have a short gonotrophic cycle, since the vector is laying its eggs in a permanent breeding sites (coastal lagoons and swamps) located in close proximity to villages and thus the human host.

The estimated length of the gonotrophic cycle (2.6 days) was longer than the estimate of the feeding cycle (2.1 days) calculated from the mark-release-recapture experiment. It is possible that the laboratory conditions (e.g., sugar deprivation, limited space, temperature, etc.) in which the gonotrophic cycle was estimated from egg development were sufficiently different from the field conditions in which the feeding cycle was measured to explain the difference between the estimates of the gonotrophic and feeding cycles. A similar study for *Anopheles albitarsis* in Brazil [37], also found a longer gonotrophic cycle (calculated from oviposition observations) than the feeding cycle (from mark-release-recapture experiments).

The *An. farauti* population in this area exhibited a single peak biting season between October and January. In Haleta the parity data followed a seasonal trend with higher parity rates occurring during peak adult densities and declining from February with lowest rates in August and November 2012 when *An. farauti* densities would begin to increase (Fig. 3) with the emergence of nulliparous mosquitoes into the adult population. This should be considered when planning vector control, with the bulk of activities completed before commencement of the peak biting season. A very similar temporal pattern and similar genetic population of *An. farauti* [18] was found in Guadalcanal [15]. A supporting study of the larval populations in Guadalcanal [38] demonstrated that larval presence and density also varied seasonally and was primarily driven by rainfall.

Historical estimates of the sporozoite rates and EIR for *An. farauti* are not available for Central Province, but are available for Guadalcanal Province (the nearest province). During the early 1990s and in the absence of vector control, EIR values as high as 1022 ib/p/y were recorded in Guadalcanal [39]. The intensified vector control programme implemented by the Ministry of Health and Medical Services over the last decade has had a substantial impact on transmission as evidenced by the greatly diminished and now relatively low EIRs estimated here in 2012 (2.5 ib/p/y) and 2013 (35.7 ib/p/y).

Despite the early and outdoor biting habits of *An. farauti*, the frequency of blood feeding by this species offers an explanation for the substantial malaria control that has been achieved by LLINs and IRS in the Solomon Islands. With each successive feeding cycle there is a multiplicative effect that increases the proportion of the total vector population exposed to insecticides. In the Solomon Islands where the annual mean temperature is ≈26 °C, the length of the extrinsic incubation period (EIP) is estimated to be 12 and 9 days for *Plasmodium falciparum* and *Plasmodium vivax*, respectively [40]. With an estimated feeding cycle of two days, *An. farauti*, will have 6 and 5 opportunities to enter a house before completion of the *P. falciparum* and *P. vivax* EIP, respectively. Although only 13 % (π_i_) of *An. farauti* will be potentially exposed to insecticides by biting late and indoors during each feeding cycle, this will cumulate in significant mortality across the multiple feeding cycles required to complete the EIP. Assuming that LLINs have the potential to kill 80 % of those mosquitoes that enter and attempt to feed on sleeping humans, this could translate into 47 and 41 % population-level mortality before *An. farauti* would be infectious to humans with *P. falciparum* and *P. vivax*, respectively.^2^ This emphasizes the fact that although the population of *An. farauti* is primarily exophagic, indoor vector control tools still provide significant control [41]. This is an important consideration, as evidence has been emerging from other anopheline populations that the proportion of feeding indoors is diminishing, such as for *An.funestus* in Tanzania [42], Benin [43] and Senegal [44] as well as *An.gambiae s.s.* in Equatorial Guinea [45].

## Conclusion

LLINs and IRS have had a significant impact on malaria transmission despite the outdoor and early biting habits of *An. farauti*, the primary malaria vector in the Solomon Islands. Here key bionomic parameters of the malaria vector, *An. farauti*, that determine the potential for transmission (i.e., vectorial capacity) and the vulnerability to control interventions were estimated. The protective effect against LLINs and IRS that *An. farauti* enjoys by virtue of biting outdoors is offset by its short feeding cycle which potentially exposes this vector 4–6 times during the course of an EIP to the insecticides in LLINs and IRS. Nonetheless, elimination will likely require vector control tools that target other bionomic vulnerabilities to suppress transmission outdoors and to complement the control provided by LLINs and IRS.

## Availability of data and materials

The datasets supporting the conclusions of this article are available in the James Cook University Tropical Data Hub repository: http://dx.doi.org/10.4225/28/56C671268CF73.

## Abbreviations

EIP: extrinsic incubation period
EIR: entomological inoculation rate
GLM: generalized linear model
IRS: indoor residual spraying
HLC: human landing catch
LLINs: long-lasting insecticidal nets
MEP: Malaria Eradication Program
WHO MPAC: World Health Organization’s Malaria Policy Advisory Committee
π_i_: the proportion of feeds on humans taking place indoors

## Footnotes

1 The π_i_ value for Guadalcanal was calculated using the raw *An. farauti* dataset from the publication and the human movement profile from Central Province presented in this paper.

2 These basic calculations account for the maximum possible efficacy of indoor vector control in the absence of any other mortality factors. The population-level mortality across multiple feeding cycles was calculated as 1−(S^F^). Where S = the proportion surviving each feeding cycle calculated as 1 – (π_i_ x 0.8); and F = the number of feeding cycles.
